# Supplementary material for: Ultra-Efficient Removal of Crystal Violet Dye Using Industrial Brine and Horn-Derived Biochar: Synergistic Action of Salting-Out/Adsorption
Source: Toxics. 2025 Nov 30;13(12):1039. doi: 10.3390/toxics13121039 (PMC12737601; doi:10.3390/toxics13121039)
Supplement: Supplementary file 1 [file toxics-13-01039-s001.zip › toxics-3990252-supplementary.pdf]

Ultra-Efficient Removal of Crystal Violet Dye Using Industrial Brine and Horn-Derived Biochar: Synergistic Action of Salting-Out/Adsorption

Asma Nouioua[a,b*], Dhirar Ben Salem[a], Abdelkader Ouakouak[c], Saadia Guergazi[a,d], Abdelouaheb Abdelli[e], Daniel Goma[f,g], Jose Manuel Gatica[f*], Hilario Vidal[f]

[a] Research Laboratory in Subterranean and Surface Hydraulics, University of Biskra, PO Box 145, Biskra, 07000, Algeria.

[b] Department of Industrial Chemistry Faculty of Science and Technology, University of Biskra, PO Box 145, Biskra, 07000, Algeria.

[c] Hydraulic and Civil Engineering Department, University of El Oued, PO Box 789, El Oued, 39000, Algeria.

[d] Civil Engineering and Hydraulic Department, University of Biskra, PO Box 145, Biskra, 07000, Algeria.

[e] Department of Quality Control, ENASEL Unit, El-Outaya Complex, Biskra, Algeria.

[f] Departamento de Ciencia de los Materiales e Ingeniería Metalúrgica y Química Inorgánica e Instituto de Microscopía Electrónica y Materiales (IMEYMAT), Universidad de Cádiz, 11510 Puerto Real, Spain.

[g] School of Chemistry and Chemical Engineering, Queen's University Belfast, David-Keir Building, Strandmillis Road, Belfast BT9 5AG, UK.

*Corresponding author E-mail:

Asma Nouioua: asma.nouioua@univ-biskra.dz

Jose Manuel Gatica: josemanuel.gatica@uca.es

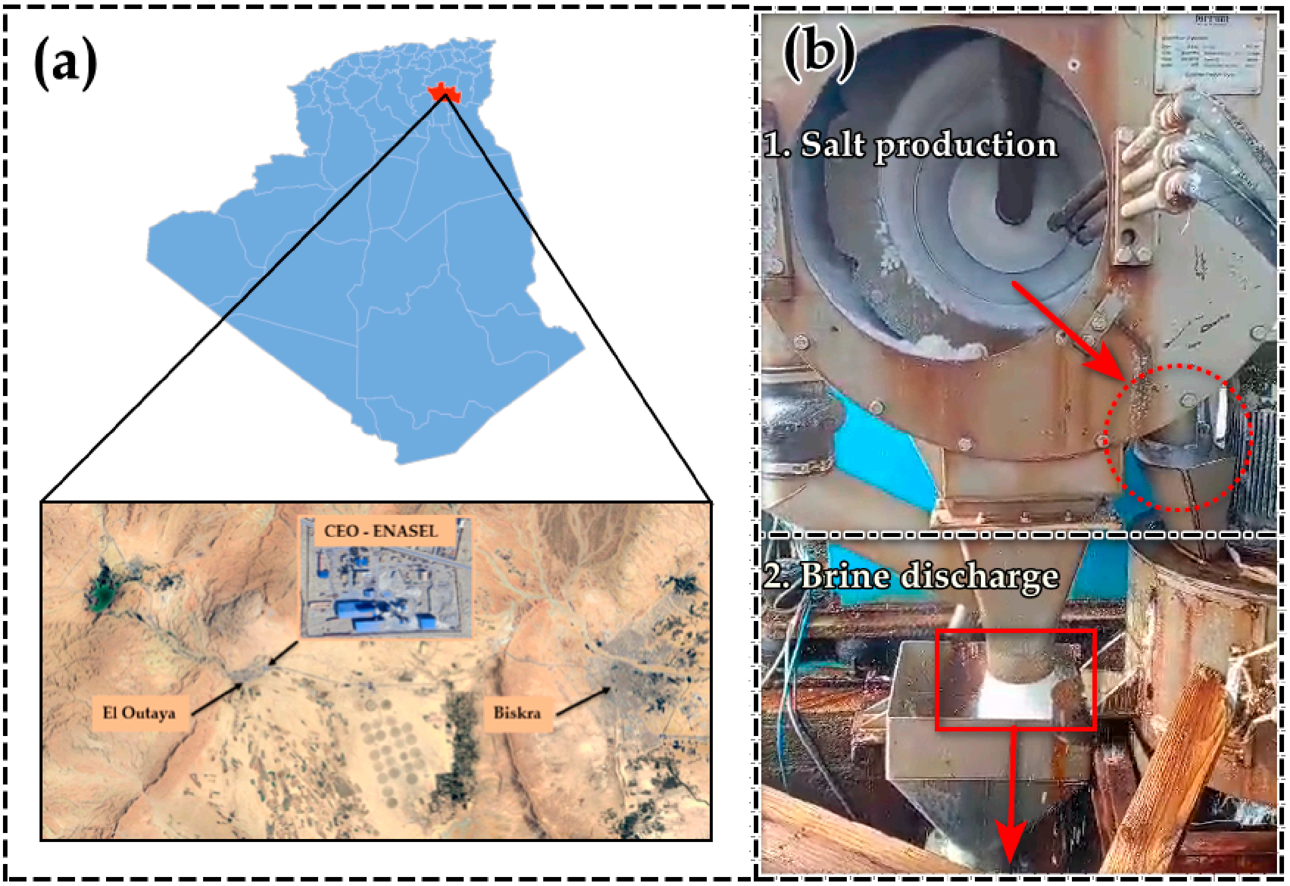

Figure S1. (a) Geographical location of brine discharge (35°01'44.6"N 5°36'06.2"E) and of the BHC-800 origin (34°51'20.2"N 5°46'11.4"E), and (b) salt production and brine discharge of the CEO-ENSAEL industry.

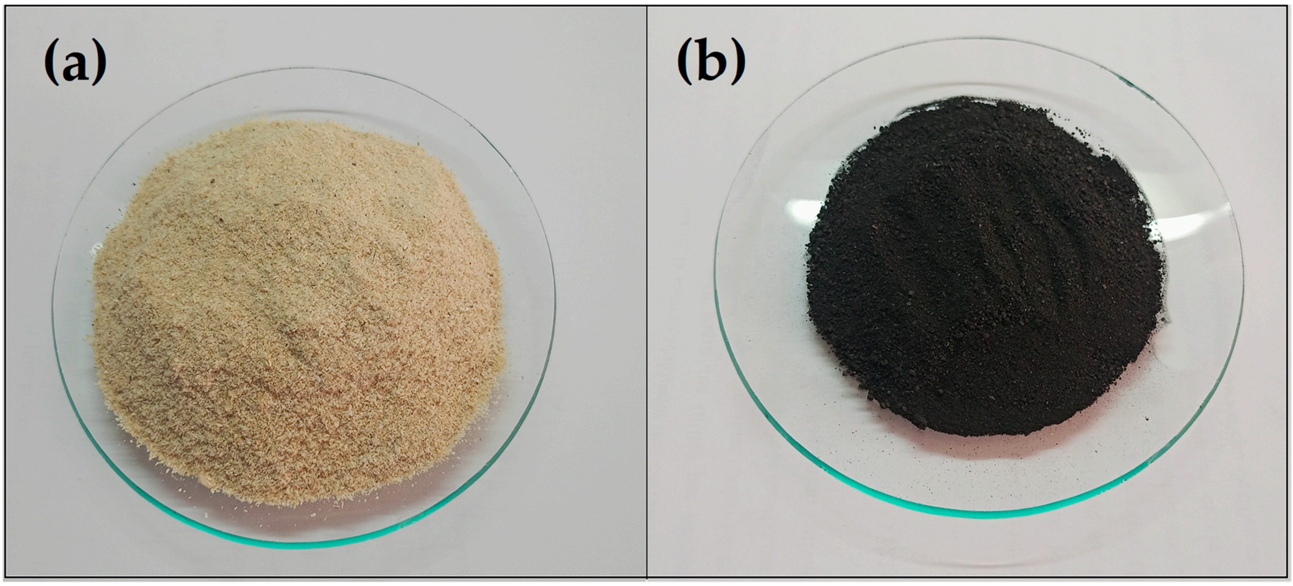

Figure S2. calves' horn: (a) biomass (0.5–1 mm), and (b) BHC-800 biochar (75–250 µm)

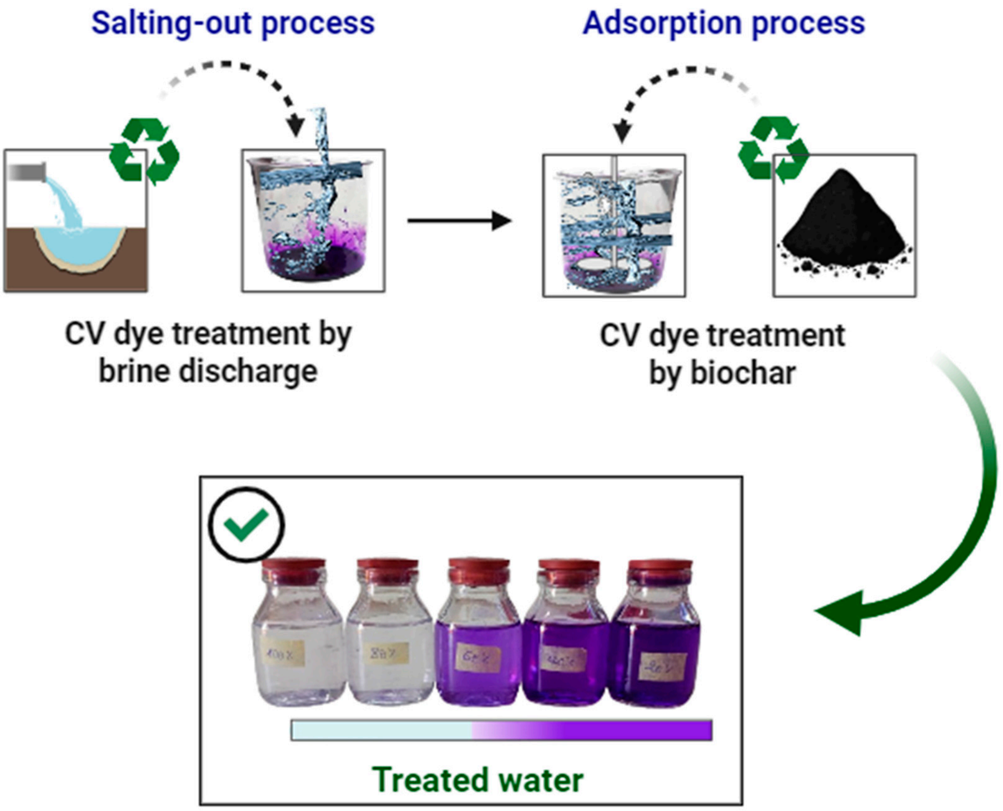

Figure S3. Schematic illustration of crystal violet dye removal via salting-out and adsorption processes

Table S1. Summary of equations, parameters, and analytical methods for crystal violet adsorption studies.

		Equation	Parameters definition	Reference
Statistical measures	The removal efficiency (R %)	$\mathrm{R}\,\% = \frac{C_o - C_e}{C_o} \times 100$ (1)	C_o : initial dye concentrations ($mg.L^{-1}$)<br>C_t : dye concentrations at any given time ($mg.L^{-1}$)<br>C_e : dye concentrations at equilibrium ($mg.L^{-1}$)<br>V : volume of the liquid phase (L)<br>m : quantity of adsorbent (g)<br>$q_{e,exp}$: adsorption capacities for the experimental at equilibrium ($mg.g^{-1}$)<br>$q_{e,model}$: adsorption capacities at equilibrium for the modeled data ($mg.g^{-1}$)<br>n : number of experimental data points<br>p: number of independent variables	[63]
	Equilibrium adsorption capacity (q_e, $mg.g^{-1}$)	$q_e = \frac{(C_o - C_e) * V}{m}$ (2)		[64]
	Instantaneous adsorption capacity (q_t, $mg.g^{-1}$)	$q_t = \frac{(C_o - C_t) * V}{m}$ (3)		
	Coefficient of Determination R^2	$R^2 = \left[\frac{\sum_i^{np} (q_{i,\exp} - q_{\exp})^2 - \sum_i^{np} (q_{i,\exp} - q_{i,model})^2}{\sum_p^{n} (q_{i,\exp} - \bar{q}_{\exp})^2} \right]$ (4)		[65]
	Standard deviation of residues (SD)	$SD = \sqrt{\left(\frac{1}{n-p}\right) \times \left[\sum \left(q_{e,\exp} - q_{e,model} \right)^2 \right]}$ (5)		
Kinetic models	PFO	$q_t = q_e (1 - e^{-k_1 t})$ (6)	q_e: Equilibrium adsorption capacity ($mg.g^{-1}$)<br>q_t : Instantaneous adsorption capacity ($mg.g^{-1}$)<br>k_1(min^{-1}), k_2($g.mg^{-1}.min^{-1}$): rates constants for PFO and PSO respectively.<br>k_{AV}: Avrami kinetic constant (min^{-1})<br>n_{AV}: fractional adsorption order corresponding to adsorption mechanism.	[66]
	PSO	$q_t = \frac{q_e^2 . k_2 . t}{1 + q_e . k_2 . t}$ (7)		
	Avrami	$q_t = q_e (1 - e^{-(K_{AV} t)^{nAV}})$ (8)		
	Elovich	$q_t = \frac{1}{\beta} \ln(\alpha \beta t) + \frac{1}{\beta} \ln t$ (9)		

			α : initial adsorption rate constant (g.mg^{-1}.min^{-1})<br>β : desorption rate constant (g.mg^{-1})<br>t: time (min)	
Isotherm models	Langmuir	$q_e = \frac{Q_{\max}.K_L.C_e}{1+K_L.C_e}$ (10)	$Q°$: maximum adsorption capacity (mg.g^{-1})<br>K_L: Langmuir constant of energy (L.mg^{-1})<br>K_F: Freundlich constant ((mg.g^{-1}). ((mg.L^{-1})^n)$^{-1}$)<br>n : intensity coefficient<br>A (mg.g^{-1}) and K_T (L.mg^{-1}): Temkin model constants.	[67]
	Freundlich	$q_e = K_F.C_e^{(1/n)}$ (11)		
	Temkin	$q_e = A\ln(K_T C_e)$ (12)		
Thermodynamic calculations	Standard Gibbs free energy change (ΔG°) (kJ.mol^{-1})	$\Delta G° = -RT\ ln\ K_L$ (13)	R : ideal gas constant (J.mol^{-1}.K^{-1})<br>T : temperature of experiment (K)<br>K_C : thermodynamic equilibrium constant<br>M_{CV} : crystal violet mass molar (mol.L^{-1})<br>$C°$: standard reference solution (mol.L^{-1})	[68]
	Standard enthalpy change (ΔH°) (kJ.mol^{-1}) and standard entropy change (ΔS°) (kJ.mol^{-1}.K^{-1})	$\ln K_e^{\circ} = \frac{\Delta S^{\circ}}{R} - \frac{\Delta H^{\circ}}{RT}$ (14)		
	Equilibrium constant K_C	$K_C \approx K_L \times M_{CV} \times C^{\circ}$ (15)		

References

63. Moussout, H.; Ahlafi, H.; Aazza, M.; Maghat, H. Critical of Linear and Nonlinear Equations of Pseudo-First Order and Pseudo-Second Order Kinetic Models. *Karbala Int. J. Mod. Sci.* **2018**, *4*, 244–254, doi:10.1016/j.kijoms.2018.04.001.

64. Ho, Y.S.; McKay, G. Sorption of Dye from Aqueous Solution by Peat. *Chem. Eng. J.* **1998**, *70*, 115–124, doi:10.1016/S0923-0467(98)00076-1.

65. Revellame, E.D.; Fortela, D.L.; Sharp, W.; Hernandez, R.; Zappi, M.E. Adsorption Kinetic Modeling Using Pseudo-First Order and Pseudo-Second Order Rate Laws: A Review. *Clean. Eng. Technol.* **2020**, *1*, 100032, doi:10.1016/j.clet.2020.100032.

66. Lima, É.C.; Adebayo, M.A.; Machado, F.M. *Kinetic and Equilibrium Models of Adsorption*; 2015; Vol. 0; ISBN 9783319188751.

67. Vargas, A.M.M.; Cazetta, A.L.; Kunita, M.H.; Silva, T.L.; Almeida, V.C. Adsorption of Methylene Blue on Activated Carbon Produced from Flamboyant Pods (Delonix Regia): Study of Adsorption Isotherms and Kinetic Models. *Chem. Eng. J.* **2011**, *168*, 722–730, doi:10.1016/j.cej.2011.01.067.

68. Tran, H.N.; You, S.-J.; Nguyen, T.V.; Chao, H.-P. Insight into the Adsorption Mechanism of Cationic Dye onto Biosorbents Derived from Agricultural Wastes. *Chem. Eng. Commun.* **2017**, *204*, 1020–1036, doi:10.1080/00986445.2017.1336090.
